# Treatment of a giant hepatic echinococcal cyst with percutaneous drainage and in vivo assessment of the protoscolicidal effect of praziquantel

**DOI:** 10.1007/s12328-021-01397-4

**Published:** 2021-04-13

**Authors:** Joachim Richter, Andreas Karl Lindner, Dominik Geisel, Giovanni Federico Torsello, Gabriela Equihua Martinez, Caroline Isner, Dirk Schürmann, Frieder Pfäfflin, Arzu Orhun, Tommaso Manciulli, Enrico Brunetti

**Affiliations:** 1 Institute of Tropical Medicine and International Health, Charité University Medicine, Berlin, Corporate Member of Free University and Humboldt University, Augustenburgerplatz 1, 13353 Berlin, FR Germany; 2 Department of Radiology, Charité University Medicine, Berlin, Corporate Member of Free University and Humboldt University, Berlin, Germany; 3 Department of Infectious Diseases and Pulmonary Medicine, Charité University Medicine, Berlin, Corporate Member of Free University and Humboldt University, Berlin, Germany; 4 Department of Plastical and Reconstructive Surgery, Charité University Medicine, Berlin, Corporate Member of Free University and Humboldt University, Berlin, Germany; 5 Department of Clinical, Surgical, Diagnostic and Pediatric Sciences, University Hospital of Pavia, Pavia, Italy; 6 Department of Tropical and Infectious Diseases, IRCCS Policlinico San Matteo Foundation Hospital, Pavia, Italy

**Keywords:** Cystic echinococcosis, *Echinococcus granulosus*, Percutaneous drainage, Praziquantel, Albendazole

## Abstract

Therapy choices for cystic echinococcisis (CE) are stage-specific: surgical, minimally invasive, medical or observation without intervention. PAIR (percutaneous aspiration, instillation of a scolicide, and re-aspiration) has been considered the treatment of choice for uncomplicated echinococcal liver cysts. However, PAIR carries the risk of toxic cholangitis or hypernatremia and that the cyst frequently refills with bile after withdrawing the catheter. We treated a patient with a giant CE 1 liver cyst with puncture drainage (PD) under albendazole coverage. Drainage enabled us to monitor the morphology of protoscolices under praziquantel (PZQ) co-medication. Protoscolices degenerated within 5 days of PZQ 50 mg/kg/d. The cyst cavity solidified with no evidence of reactivation or secondary spread. Percutaneous treatments can replace surgery in a significant number or cases with hepatic CE. PD allows to assess microscopically the viability of protoscolices under co-medication with PZQ–albendazole and to avoid the instillation of topical scolicides.

## Introduction

**Fig. 1:**
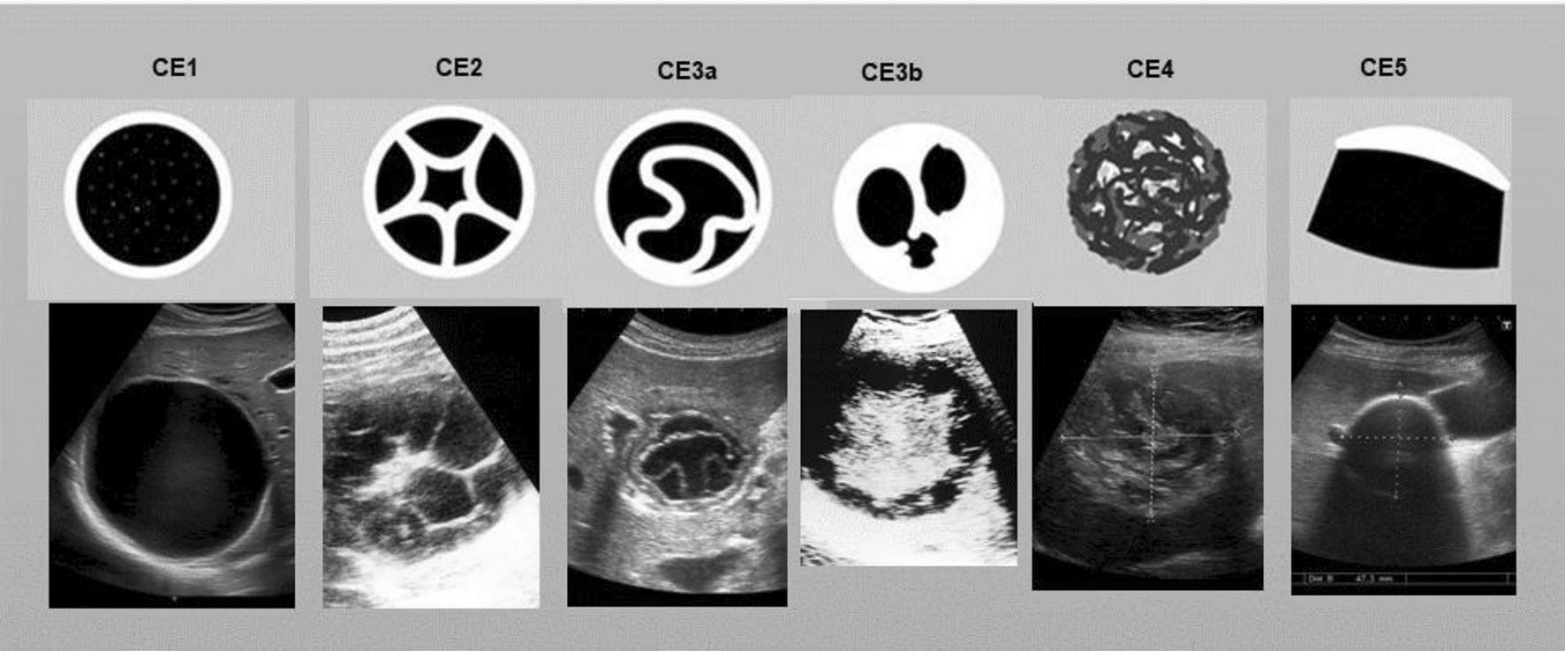
WHO-IGWE-classification of hepatic echinococcal cysts^1^

The clinical management of cystic echinococcosis (CE) is complex, as the various manifestations of this disease with multiple variables such as cyst number, dimension, stage, anatomical location make a systematic analysis difficult. An expert group convened by the World Health Organization has proposed a more rational approach to CE through a standardized ultrasound classification of echinococcal cysts [1, 2]. Hepatic echinococcal cysts were classified according to their morphology and activity (Fig. 1) [1, 2]. This enables a stage-appropriate treatment decision. Current therapy options include surgical procedures, percutaneous therapy, conservative therapy with antihelminthics and observation without intervention (“watch & wait”) of inactive cysts. If invasive interventions are planned, these must be carried out under an anti-helminthic coverage with a benzimidazole, preferably albendazole [1–5].

## Case report

A 30-year-old Kurdish patient from Syria visited the Institute for Tropical Medicine in Berlin because of suspected CE of the liver. Ten days earlier, a cholecystectomy had been performed because of acute gallbladder hydrops due to the compression of the cystic duct by a large liver cyst. The surgeons decided against removal of the large cyst during the emergency surgery. When the patient visited our outpatient department, the imaging showed a typical CE1 liver cyst with a characteristic external echogenic wall and a size of 15.5 × 9.2 × 8.1 cm (Fig. 1, 2a). The CE was confirmed by positive specific antibody test results: *Echinococcus granulosus* EIA IgG 92 AU (antibody units; norm: < 15; Euroimmun. Lübeck, Germany); HAT IgG 1: 1024 (Norm: 1: 32–1: 64; Siemens, Erlangen, Germany). Secondary extrahepatic dissociation of CE was excluded by abdominal CT and imaging of the lungs and brain.

**Fig. 2:**
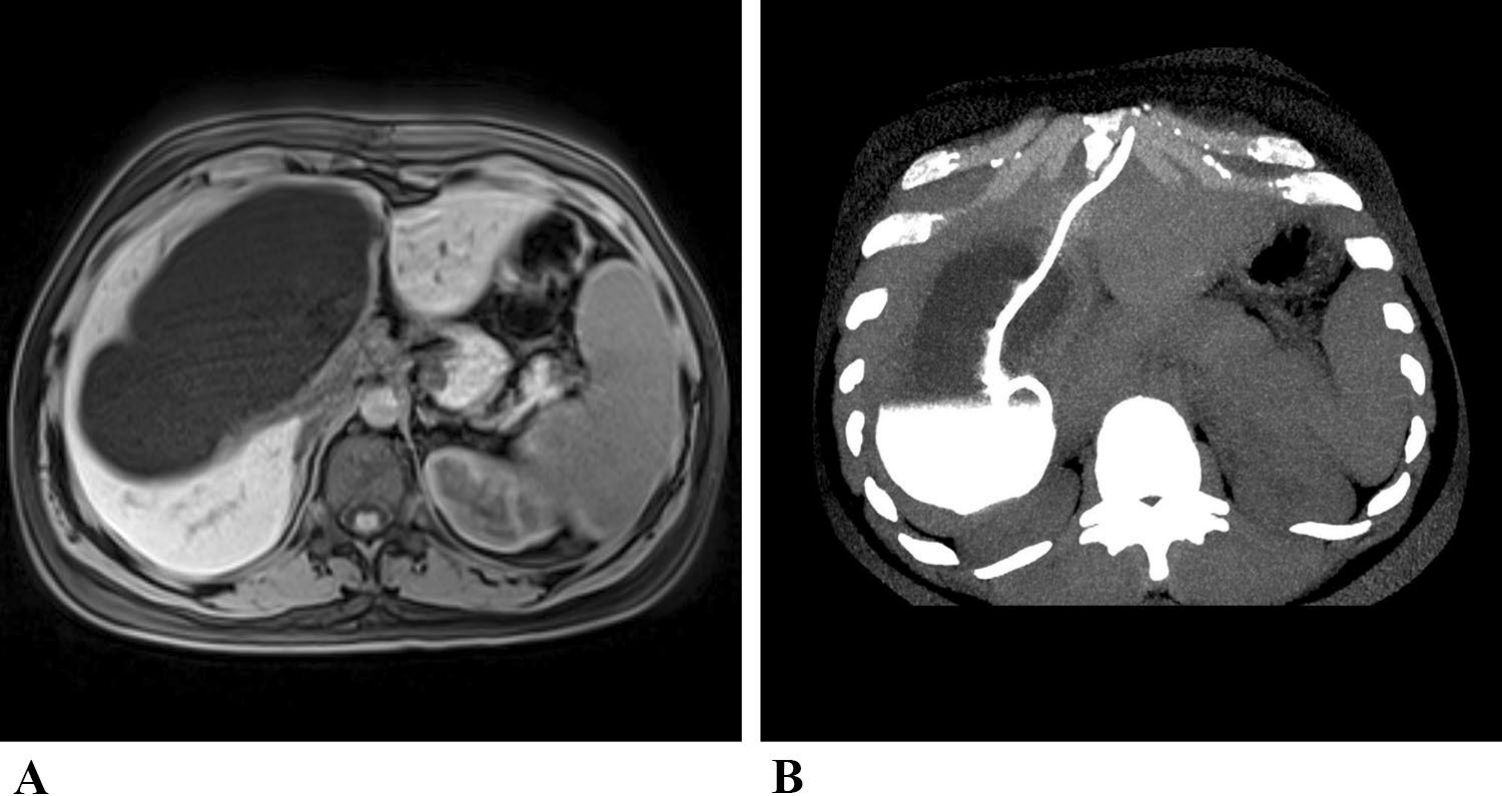
**a** Magnetic resonance imaging of the giant hepatic echinococcal cyst; **b** Computed tomography reconstructed in maximum intensity projection (MIP) after catheter placement. The cyst is partially filled with contrast medium

**Fig. 3:**
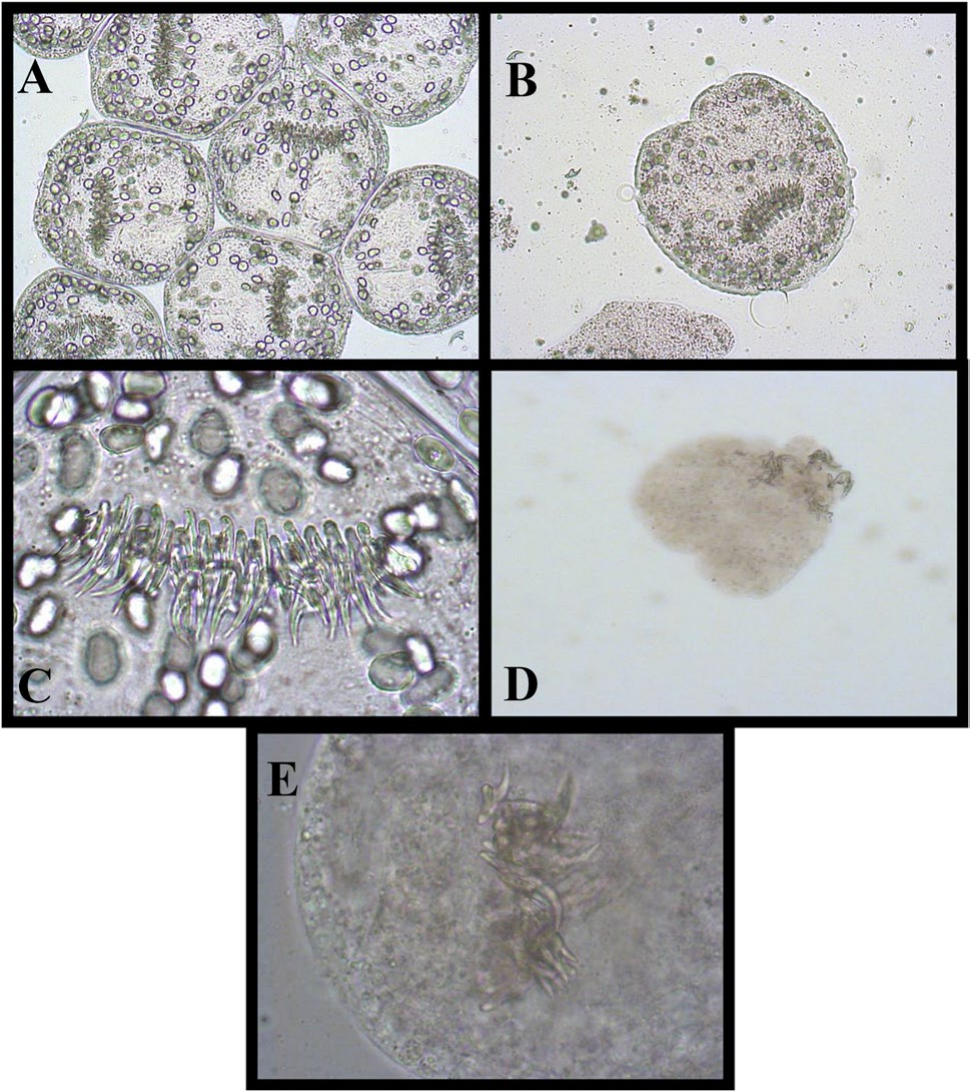
Microscopy of the cyst fluid: **a**.multitude of viable protoscolices at puncture, 21 days after starting albendazole monotherapy; **b** single protoscolex; **c** intact rostellum with hooklets; **d** degenerating protoscolex; **e** disintegrated rostellum five days after adding praziquantel

Albendazole (ABZ) therapy was started with a standard dose of 2 × 400 mg/day. After carefully examining all treatment options, we decided on a minimally invasive approach: a lockable 12-F drainage catheter (multi-purpose drainage catheter, Cook Inc., Bloomington, USA) was percutaneously inserted into the liver cyst using a Seldinger approach inserted into the liver cyst and fixed to the skin with a suture (Fig. 2b). Allergic events did not occur either during or after the procedure. PZQ was added to the ABZ medication in a daily dose of 50 mg/kg body weight. More than 1500 ml of fluid was drained from the collapsed cyst in the following days. As a result, the cyst membrane collapsed and the cyst morphology converted into CE3a. Microscopy of the cyst material aspirated through the catheter on the first day showed a large number of morphologically intact protoscolices despite the previous 21-day ABZ administration. Within 5 days after adding PZQ to ABZ, the protoscolices degenerated completely. The hooks had lost their morphological alignment in the rostellum, (Fig. 3). The ABZ-sulfoxide serum concentration was found < 0.43 mg/l, a concentration below the defined efficacy level of 0.5–1.7 mg/l. This confirmed the protoscolicidal effect of PZQ in vivo*,* although PZQ concentration in the cyst fluid and serum could not be determined for logistic reasons.

**Fig. 4:**
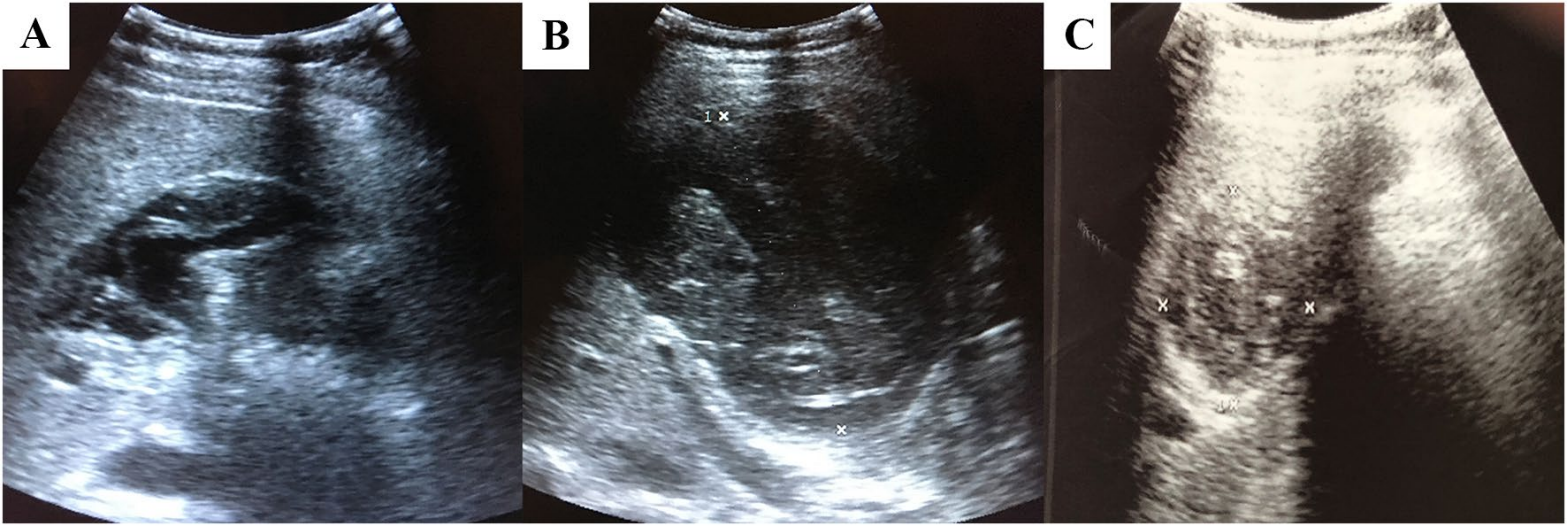
**a**–**c** Abdominal ultrasonography: subsequent evolution of the liver cyst after aspiration and combined albendazole—praziquantel therapy. **a** after having 13 withdrawn 1500 ml of cyst fluid; **b** one week after removal of the catheter; **c** twenty-three months after PD

Another 4 months later, the cyst cavity had narrowed to 5.0 cm and solidified into a CE4 cyst until last seen 23 months later. Thus, the ABZ therapy was ended. During the 23-month monitoring period to date, there has been no evidence of a relapse or occurrence of secondary CE (Fig. 4). The antibody serum concentration decreased to EIA IgG 20 AU and the specific HAT test turned negative.

## Discussion

Surgical intervention is still the first choice for complicated abdominal cysts and hepatic cysts in stage CE2 and CE3b. This also applies to cysts with less common extra-abdominal locations such as heart, lung, CNS and bone CE, that often do not respond to conservative therapy [1, 2, 4, 6, 7]. Surgical therapy must be carried out under benzimidazole (BMZ), such as ABZ or mebendazole (MBZ), coverage to avoid secondary echinococcosis after accidental leakage of cyst fluid into the peritoneum [1, 2, 5, 8].

Conservative BMZ therapy is the best option for treating small cysts less than 5 cm in diameter in stage CE1 and CE3a or when the CE already affects several organs [1, 2, 4, 9]. If ABZ is not tolerated, MBZ can be given instead.

Larger uncomplicated CE1 and CE3a cysts can be treated with percutaneous treatments depending on their size [1, 2, 4, 9]. Puncture, aspiration, topical instillation of a scolicide and re-aspiration (PAIR) has been recommended as the first-line therapy option for simple CE1 cysts. 98% ethanol or 20% saline solution are most commonly used as scolicidals [1, 2, 4, 9]. The risk of anaphylaxis when puncturing a hepatic CE cyst under ABZ coverage appears to be comparable to that of surgery [10–13]. On the other hand, topical intracystic scolicidals bear several risks that increase in direct proportion to the cyst size: highly concentrated ethanol can cause severe chemical cholangitis when accidental leakage into the biliary system occurs [2, 4]. Hypertonic saline has been reported to sometimes cause severe or even fatal hypernatremia [14–16]. Furthermore, in the case of a PAIR, in which the catheter is usually removed immediately after aspiration, bile frequently flows into the cyst cavity, which can falsely give the impression of a relapse and solidification of the cyst is delayed. For these reasons, we opted for a percutaneous drainage (PD) which has been recommended for “giant” cysts > 10 cm [1, 17, 18]. Instead of an intracystic instillation of a scolicide, we added the oral scolicidal anti-helminthic drug praziquantel (PZQ) [1, 17–22]. In humans, PZQ inhibits the vesicular development of protoscolices and thus prevents the formation of secondary cysts. PZQ also inhibits cyst differentiation and the development of the fibrous adventitial layer. PZQ is considerably more effective against intracystic protoscolices than ABZ in vitro and in animal models [3, 17–22]. The data on whether PZQ alone is also sufficiently protoscolicidal in vivo*,* or whether this is partly due to a synergy with ABZ, are contradictory [19–22]. Therefore, the data situation does not yet allow a general recommendation for the PZQ dosage and duration of therapy to be formulated [22]. In the present case, the drainage was left in place until the cyst collapsed completely and only minimal amounts of fluid emerged from the catheter. This enabled us to examine the cyst aspirate microscopically and to assess the morphology of the intracystic protoscolices at any time**.** Since ABZ pretreatment without PZQ had not affected the protoscolices and the ABZ serum concentration remained below the specified therapeutic serum concentration 2 days after the additional administration of PZQ, we assume that the protoscolicidal effect was mainly due to PZQ. Repeated microscopy of the cyst fluid documented the degeneration of the protoscolices in vivo, which occurred within 5 days after adding PZQ for at a dose of 50 mg/kg body weight/day an observation essentially confirmed by two other cases that we treated according to the same protocol (not listed here).

Cystic echinococcosis (CE) is a worldwide widespread zoonosis that has been included by the WHO into the list of Neglected Tropical Diseases (NTD). In this context, CE is classified as an "orphan disease" [24]. CE mainly occurs in areas where slaughter is not controlled. Dogs become infected when they have access to infected offals. Human infection is acquired through direct or indirect contact with dog feces and subsequent ingestion of taenia eggs [4]. Measures to prevent transmission include hygienic measures, the physical separation of dogs and farm animals, controlled slaughter of livestock, boiling offal before it is fed to dogs and regular PZQ deworming of dogs. No final recommendation can yet be made regarding the effectiveness of sheep vaccines. Control measures are effective but take time to achieve disease control and their cost-effectiveness depends on the burden of disease. [4, 25]. A Canadian study has shown that these measures are cost-effective at least when the prevalence of CE exceeds 13/100,000 people [26].

In non-endemic areas, CE mainly affects refugees and immigrants from rural areas, where the prevalence of CE can exceed 5% [27]. In terms of treatment, the stage-matched approach to liver cysts allows a rational choice that avoids ineffective (ABZ or PAIR for CE2 and CE3b cysts) or unnecessary over-treatment. Any surgery on uncomplicated inactive cysts is potentially dangerous and a waste of medical resources [1, 4, 23, 28].

Concluding, CE is a global zoonosis that requires a complex, stage-appropriate, multidisciplinary therapeutic approach. Optimal treatment of hepatic echinococcosis depends on the WHO staging through imaging (Fig. 1). Surgical therapy is usually necessary for CE2 and CE3b liver cysts. CE4 and CE5 liver cysts are observed without intervention. Unilocular echinococcal cysts (CE1, CE3a) can be treated conservatively or minimally invasively: for CE1 cysts < 5 cm, a purely drug-based treatment attempt with a BMZ can be made, for CE1 cysts of 5–10 cm a PAIR is the first choice, for CE1 cysts > 10 cm a PD is indicated. The protoscolicidal PZQ can replace potentially harmful topical protoscolicidals. When a PD is performed, the microscopic monitoring of the larvae in the cyst aspirate allows the individual optimal PZQ treatment dose and duration to be determined. Conservative and percutaneous therapy can replace invasive surgical interventions in a significant number of cases and thus reduce the length of hospital stay, complications and intervention costs, which is of particular importance in resource-poor endemic areas. In the present case, the cumulative length of hospital stay was 9 days, compared to an average length of hospital stay of a surgically treated patient of 20 days [28].
